# Psychiatry

**Published:** 1905-01-07

**Authors:** 


### PSYCHIATRY.

Treatment of Insanity by Hypnotics.—Professor Diefendorf,[1] of Yale University, states that it is very important that relief should be given to the distressing insomnia which is characteristic of and heralds the early stages of many forms of insanity. Insomnia of a less intense character is met with after the insanity or psychosis is well established, and this also demands special treatment. These two kinds of insomnia differ radically; the insomnia of pro-dromal insanity is one which is almost invariably present; it varies from mild sleeplessness to intense and distressing insomnia, and is usually associated with cephalic paraesthesiæ from the earliest period, *e.g.* feelings of "pressure" and "constriction" of the skull, "fulness" of the head, vague apprehensions with suicidal thoughts, and fear of "loss of mind." This period of insomnia is very often aggravated by dreams of the most distressing character. Accompanying the insomnia are progressive loss of appetite and of body-weight, reduced sexual instinct, which causes a foreboding of impotence, or, *per contra*, increased sexual desire. The treatment consists in correcting gastro-intestinal catarrh, lithæmic and gouty states, removing the patient from sources of noise, worry, and irritation, and placing him in a quiet and restful environment. Warm baths and massage are also useful in promoting sleep. Of drugs, says Dr. Diefendorf, there is no ideal one, but a safe and generally useful preparation is potassium and sodium iodides (5 grains each), with 3 to 4 grains of chloral hydrate, given in syrup of orange-peel and water. Trional is preferable to sulphonal because of its more rapid action and elimination and the absence of cumulative effect. The sulphonal should be given in hot milk, the dose being 10 to 25 grains on alternate nights and regularly for not more than two weeks. The dose should then be stopped for a few days and recommenced if insomnia persists or returns. The insomnia of a well-established mental disease is usually less urgent than the form described above in its demands for treatment. It is not so universally present as in the early stages of insanity, and when it does occur is usually associated with an exacerbation of the insanity itself. The insomnia is here associated with particular symptoms of the psychosis, viz., motor excitement, hallucinatory delirium, and delusions of fear. The combination of bromides and chloral mentioned above is the best remedy for such attacks, and the use of the wet pack is often found to allay the symptoms of excitement and delirium, though it must be applied with caution. If the patient has a weak, dilated, or fatty heart, paraldehyde should be given rather than chloral. Next in efficiency to paraldehyde is chloralamide, which can be given with safety when cardiac and vascular symptoms exist. Chloralamide must not be administered in alkaline or hot media. "If all of these hypnotics fail, one will usually succeed with a combination of ammonium bromide, 20 grains; chloral hydrate, 15 grains, and liquor morphinæ bimeconatis, 10 minims. Prolonged deep sleep is not to be expected, as this comes only with general improvement." The insomnia of the depressive phases of dementia præcox and of the manic-depressive insanities are best ameliorated by the use of the cold or warm pack. For the extreme insomnia of infectious delirium, and of acute delirium with tendency to collapse, alcohol (whisky or brandy) is the best remedy and should be given along with liquid nourishment (milk with eggs beaten up), either by the mouth or by the œsophageal tube. In similar cases warm baths are useful. Of the newer drugs in the treatment of insomnia of insanity veronal is as good as trional.

[1] Jour. of the Amer. Med. Assoc., Nov. 19, 1904.

*(To be continued.)*

